# Efficacy of hyaluronic acid gel and photobiomodulation therapy on wound healing after surgical gingivectomy: a randomized controlled clinical trial

**DOI:** 10.1186/s12903-023-03519-5

**Published:** 2023-10-27

**Authors:** Basma Khalil Yakout, Fatma Ramzy Kamel, Maha Abd El-Aziz Abou Khadr, Lamia Ahmed Hassan Heikal, Gillan Ibrahim El-Kimary

**Affiliations:** 1 Department of Oral Medicine, Periodontology, Oral Diagnosis and Oral Radiology, Faculty of Dentistry, Alexandria University, Champolion St. Azarita, Alexandria, 21521 Egypt; 2 Department of Pharmaceutics, Faculty of Pharmacy, Alexandria University, Alexandria, Egypt

**Keywords:** Hyaluronic acid gel, Photobiomodulation therapy, LLLT, Surgical gingivectomy, Wound healing

## Abstract

**Background:**

Surgical gingivectomy can be considered the gold standard treatment for gingival enlargement. The healing of wound site after gingivectomy occurs slowly by secondary intention. To accelerate the wound healing process, several studies have been conducted evaluating the effect of various treatment modalities. Photobiomodulation therapy (PBMT) was proposed to provide minimally invasive and painless treatment as well as to decrease discomfort of the patient following the surgical process. Another factor that is expected to improve the healing after surgery is topical application of chemotherapeutic agents such as Hyaluronic acid (HA). This study aims to assess the effect of topically applied HA gel after PBMT on the healing of wound site after surgical gingivectomy.

**Methods:**

This randomized controlled clinical trial included twenty-six surgical gingivectomy wound sites, equally divided into two groups, Group-I (test group): the surgical sites after gingivectomy were irradiated with a diode laser (980 nm, 0.2 W) then covered by 2% HA gel loaded in a special custom-made soft transparent tissue guard appliance for each patient. Group II (control group): the surgical sites were irradiated with a diode laser (980 nm, 0.2 W) only. Wound healing was assessed subjectively by Landry healing index on the 3rd, 7th, 14th and 21st days after surgery, and pain perception was assessed by the patients using visual analog scale (VAS) throughout the 21 days of the follow up period. Comparisons between the two study groups were performed using Mann-Whitney U test, while comparisons between different time points were performed using Friedman test. Significance was inferred at p value < 0.05.

**Results:**

By the end of the follow-up period, surgical sites of the test group showed excellent healing compared to the control group. There were no significant differences in VAS scores between both groups (p > 0.05).

**Conclusions:**

Application of 2% HA gel as an adjunctive to PBMT was found to have significant clinical effects and higher power of repair among test group when compared to that achieved by PBMT alone in control group.

**Trial registration:**

This study was retrospectively registered on ClinicalTrials.gov and first posted on 28th of March 2023 with an identifier number: NCT05787912.

## Background

The etiology of gingival enlargement is associated with many factors including inflammation, drug use, systemic diseases, and neoplastic conditions [1]. It is characterized by an abnormal overgrowth of the connective tissue with increased number of cells. This condition affects patient’s esthetics especially if present in the anterior maxillary or mandibular areas and leads to plaque accumulation. In these cases, non-surgical phase I therapy alone may not help in reducing gingival enlargement and inflammation [2].

Management of periodontal disease must include consideration of re-establishment of the physiologic gingival architectural form. In case of gingival enlargement, the therapeutic technique to accomplish this may be done either by gingivoplasty or gingivectomy procedure [3]. This results in a favorable environment for gingival healing and restoration of physiologic gingival contour. Gingivectomy can be performed using scalpels, lasers, electrosurgical units, and chemicals such as 5% paraformaldehyde or potassium hydroxide. Surgical gingivectomy may be performed either using gingivectomy knives or surgical blades [4].

The wound site after gingivectomy and gingivoplasty operations heals by secondary intention [5]. It takes about four weeks for complete epithelialization and about seven weeks for connective tissue maturation, which makes the wound healing process after scalpel gingivectomy a relatively slow phenomenon [6]. In order to enhance the process of wound healing, numerous research studies have been carried out to assess the impact of different topical treatments and systemic antibiotics. These investigations have documented enhanced secondary intention wound healing outcomes following the application of various agents [1, 2, 7].

Photobiomodulation (PBM) is the direct application of light to stimulate cell responses in order to promote tissue healing, reduce inflammation and induce analgesia [8]. In older literature, the term low level light/laser therapy (LLLT) was used [9]. The use of photobiomodulation therapy (PBMT) for healing of wound site after surgical gingivectomy has been reported in many studies with improved and accelerated post-operative healing [9–14].

The optimal wavelengths for PBM are in the red or near-infrared spectrum in the range of 600 to 700 nm and 780 to 1100 nm [9]. The output power can vary widely from 1 mW up to 500 mW in order not to allow thermal effects [15]. However, the effects on exposed oral soft tissue wounds and the most suitable laser characteristics and settings to promote the healing of these types of wounds have not been specified to date.

Another factor that is expected to improve healing after surgery is the application of topical agents. Hyaluronic acid (HA), also known as hyaluronan, is one of the most recently used topical chemotherapeutic agents [16]. It was observed that HA has an anti-edematous and anti‑inflammatory effect acting as a scavenger which drains metalloproteinases, prostaglandins and other mediators which promote inflammatory activities [17].

It is conceivable that hyaluronan administration to periodontal wound sites could achieve comparable beneficial effects in wound healing [18]. Romeo et al. [19] observed faster wound healing via secondary intention in laser-induced wounds after the application of HA-based compound. Furthermore, Yildirım et al. [20] observed an accelerated palatal wound healing and decreased post-operative pain and discomfort with topical application of HA on the palatal donor sites.

As per our knowledge, no study in the literature has been conducted reporting the healing outcome of surgical gingivectomy wound areas when combining both, HA gel topical application with PBMT. Based on the previously mentioned properties of HA, the aim of the current study was to evaluate the effect of topical pure 2% HA gel after PBMT on the healing of surgical gingivectomy sites.

## Materials and methods

### Ethical approval and informed consent

The study protocol was conducted following the ethical guidelines of Research Ethics Committee of Faculty of Dentistry, Alexandria University (IRB No. 0001056 – IORG 0008839) in accordance to the principles of the modified Helsinki code for human clinical studies (2013) [21]. The purpose and nature of the study were explained, and the participants gave written Informed consent to participate in the study prior to any intervention.

### Study design

**Fig. 1:**
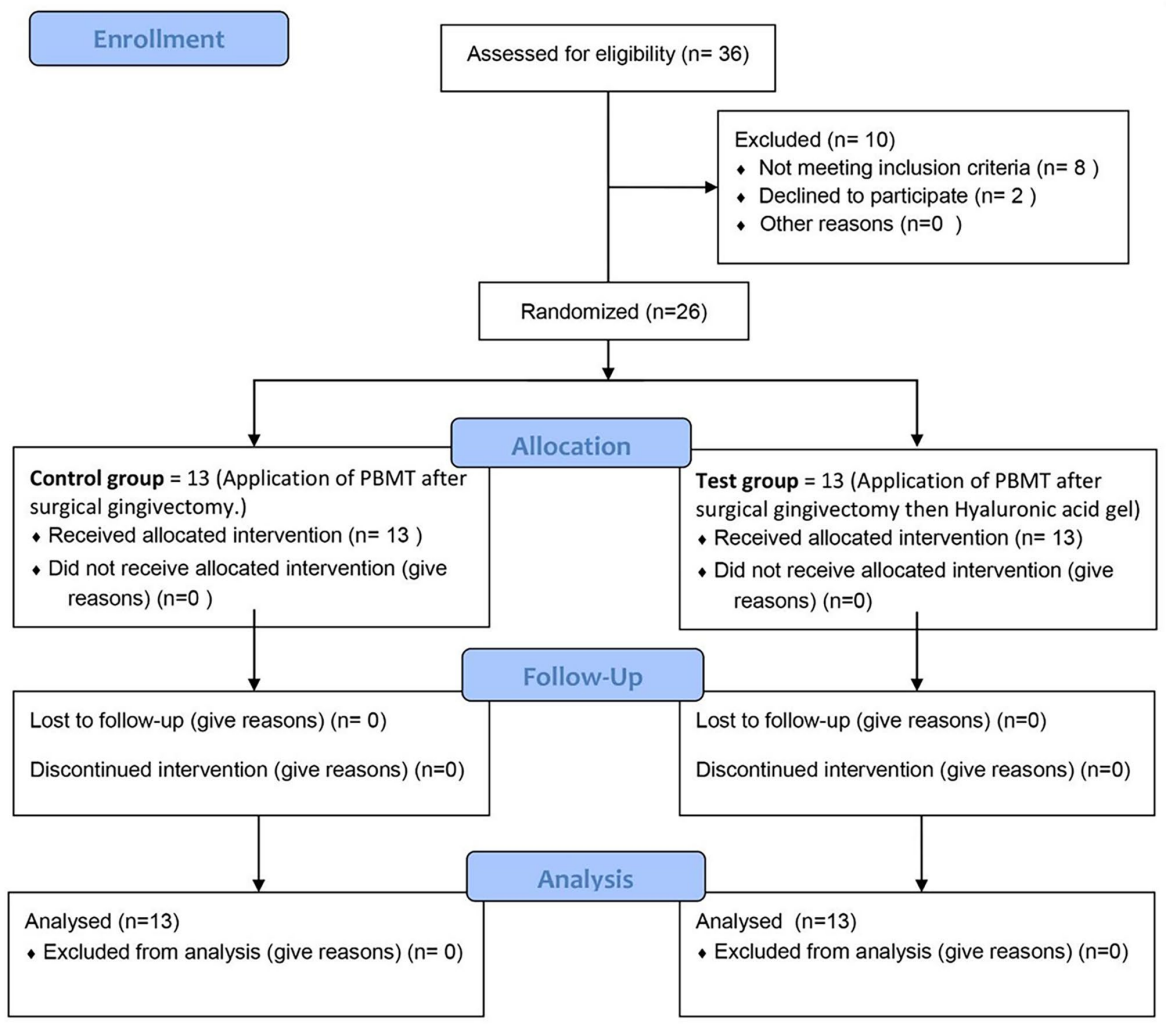
Flow chart of participants. PBMT, Photobiomodulation therapy

This randomized controlled clinical trial has been conducted following CONSORT^®^ guidelines [22]. It was carried out between February 2022 and February 2023 at the department of Oral Medicine, Periodontology, Oral Diagnosis and Oral Radiology, Faculty of dentistry, Alexandria University, Alexandria, Egypt. (Fig. 1)

### Sample size calculation

Sample size was based on 95% confidence level to detect differences in wound healing after gingivectomy between PBMT alone or with the application of topical hyaluronic acid. Reddy et al. [2] reported a mean ± SD difference in Gingival Enlargement Index (GEI) after 6 weeks of using LLLT and hyaluronic acid = 1.30 ± 0.48, and 1.20 ± 0.42. The calculated mean ± SD difference = 0.10 ± 0.45 and 95% CI= -0.32, 0.52. The minimum sample size was calculated to be 12 per group, increased to 13 to make up for cases lost to follow up. The total sample size required = number of groups × number per group = 2 × 13 = 26 [23].

### Patient selection

Twenty-six patients requiring gingivectomy procedure and met the study inclusion criteria were included in this study. They were recruited from the Faculty of Dentistry, Alexandria University outpatient clinic at the Oral Medicine, Periodontology, Oral Diagnosis and Oral Radiology department.

Inclusion criteria for the study were as follow:

- Chronic inflammatory gingival enlargement grade 2 and 3 according to the gingival overgrowth index by Miller and Damn [24].
- Adequate amount of keratinized tissue.
- Attachment loss = 0.
- Average age between 18 and 40 years.
- Well educated patients as post-operative instructions need to be followed precisely.

While we excluded: Patients with history of smoking, pregnant women, patients with bad oral hygiene, patients who have any known systemic disease that interfere with performance of surgical gingivectomy or periodontal wound healing and patients who have any previous adverse reactions to the products (or similar products) used in this study.

### Randomization and blinding

Participants were randomly allocated into two groups: test and control. Using permuted block randomization technique, the allocation sequence was generated using computer-generated random allocation software [25] where participants were allocated in blocks of 4. The allocation sequence were then sealed in opaque envelopes by a trial independent individual, who was responsible for keeping the envelopes and unfolding them only at the time of treatment [26]. Blinding of the operator and participants could not be performed due to the differences between the two techniques. However, the outcome assessor was blinded to the participants’ group allocation. All surgical gingivectomy procedures were performed by a single operator, while the outcome assessment was performed by another examiner. Calibration on Landry’s healing index scores was performed for a single examiner, intra-examiner reliability was calculated and intraclass correlation coefficient (ICC) was 0.91 indicating excellent reliability [27].

### Hyaluronic acid gel

The gel was prepared at the faculty of Pharmacy, Alexandria University, Egypt. To prepare 10 g of HA gel (2%), 0.2 g of high molecular weight (HMW) HA (Sigma, 99% purity) was dispersed in 10 mL deionized water at low pH (3.5) while stirring with a magnetic stirrer (IKA, UK) at 600 rpm for 25^o^C, preservative was dissolute in the mixture. pH is then adjusted to 6.8 where a clear viscous gel is formed. The prepared gel then was sterilized [28, 29].

Gel characterization was done as following: Viscosity measurement, Spreadability test and mucoadhesiveness, Invitro release (Using dialysis diffusion method) and stability testing [30]. Also, important pre-clinical safety tests were performed like Sterilization Validation Test, Allergenicity test and Toxicity test [31].

The concentration of HA gel used in the current clinical trial was 2% based on the final report of the safety assessment of hyaluronic acid, potassium hyaluronate, and sodium hyaluronate published in the International Journal of Toxicology [32]. No side effects were reported by any of the participants regarding to using this gel throughout the study.

### Pre-surgical preparation

Preoperatively, all patients underwent a thorough professional mechanical plaque removal (PMPR), with oral hygiene instructions. After the completion of nonsurgical phase I therapy, patients were recalled after four weeks for tissue re-evaluation. Patients with a full mouth O’Leary plaque index of less than or equal to 10% and a full mouth Gingival Index of zero were eligible to participate in the surgical procedure [33].

Primary impression was taken for test group patients by alginate material (Zhermack Alginate Hydrogum 5 Impression material) to fabricate the transparent tissue guard appliance.

### Surgical procedures

All surgeries were performed by the same operator. After administration of local anesthesia (4% articaine hydrochloride and 1:100000 adrenaline) an external bevel gingivectomy was started. The gingival pockets were examined with UNC periodontal probe (Nordent-USA) and marked with a pocket marker, then Kirkland knife (Medesy®-Italy) was used for external bevel incision and Orban knife (Medesy®-Italy) was used for releasing the interdental tissue. The remaining gingival fragments were removed using periodontal curette and micro-surgical scissors.

**Fig. 2:**
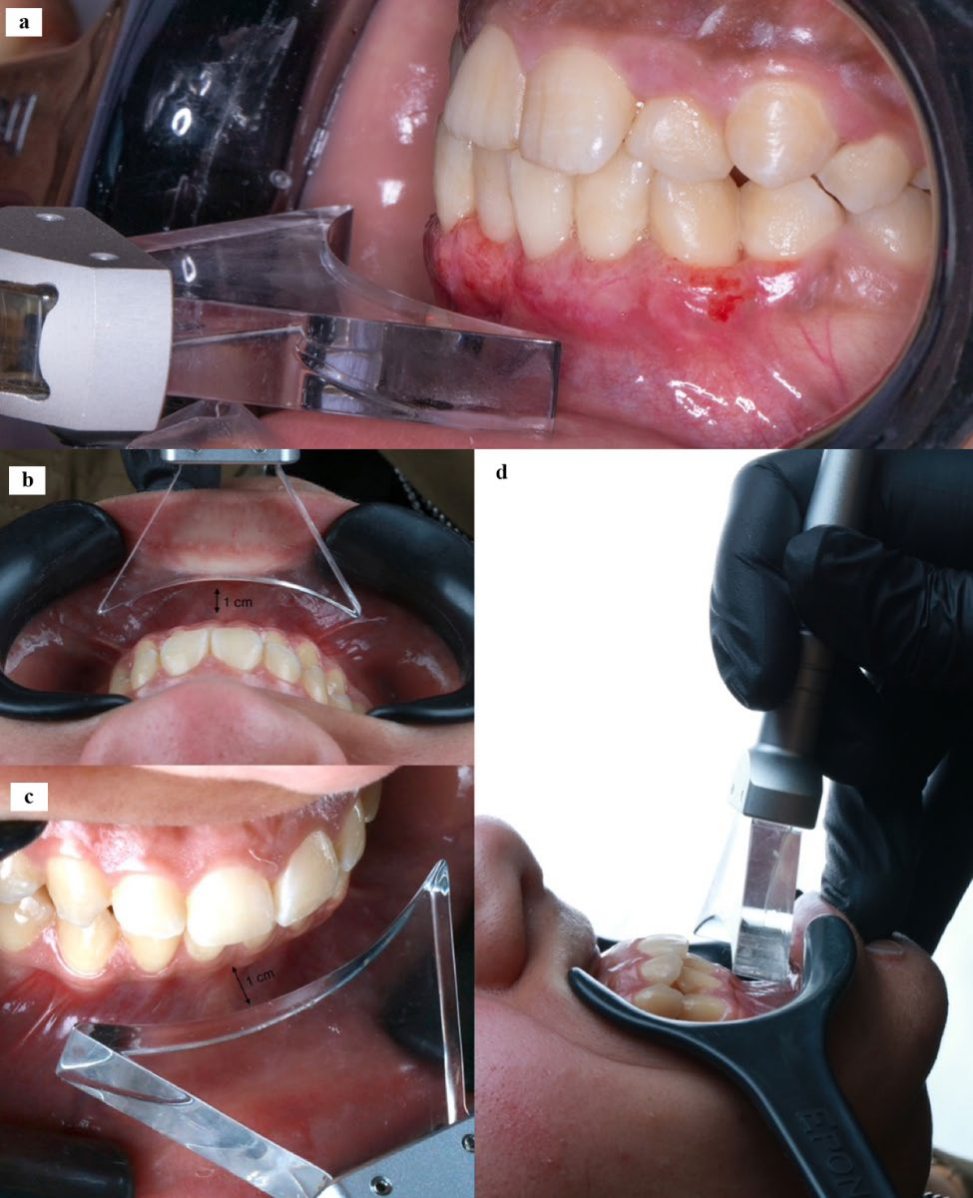
The delivery tip of the diode laser device applied perpendicular to the gingival tissue surface at 1 cm distance. **a**. shows the application of PBM on the 3rd day after surgery. **b,c**, and **d** shows the applied tip at exact distance from 12 o’clock view, frontal view and lateral view respectively

**For group I**, after hemostasis, the surgical sites of the test group were subjected to laser irradiation using diode laser. The Diode laser used in this study for PBMT was Doctor Smile diode laser – Manufacturer LAMBDA SpA –Italy. The parameters were settled on wavelength = 980 nm, Power = 0.2 W, Time = 60 s [8], The delivery tip diameter = 4 cm X 1 cm applied perpendicular to the gingival tissue surface at 1 cm distance (Fig. 2), the radiation was in continuous wave mode and the radiant exposure = 3 J/cm2. All the selected parameters (Table 1) were within the range of the typical parameters for PBMT published in 2014 by Carroll et al. [8]. The treatment intervals for each patient in both groups (test and control) were: postoperatively at the day of surgery, day 3, day 7 and day 14. These are the same intervals for radiation used in many studies that examined PBMT on gingivectomy wound healing [1, 11, 34]. During these initial stages of wound healing, there are formation and proliferation of new blood vessels and fibroblasts.

**Table 1:** Parameters used for PBMT

Wavelength (nm)	980 nm
Power (mW)	200 mW
Irradiation time (seconds)	60 s
delivery tip diameter (cm)	4 × 1 cm
power density (mW/cm^2^)	50 mW/cm^2^
Energy density (J/cm^2^)	3 J/cm^2^
Total amount of energy (J)	12 J
Application distance (cm)	1 cm (non-contact)
Mode	Continuous wave
Frequency of treatment	4 times (immediately after surgery and at days 3, 7 and 14)

**Fig. 3:**
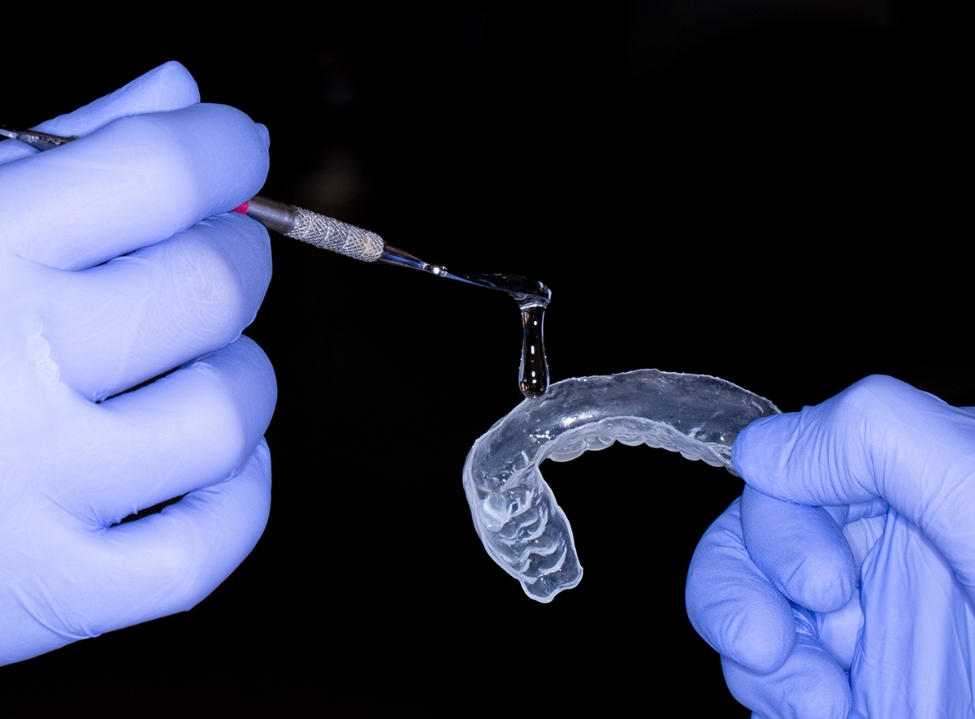
Topical Hyaluronic acid gel (2%) loaded in a custom-made transparent soft tissue guard appliance

Following PBM, the wound was covered by topical HA gel at a concentration of 2% HMW loaded in a special custom-made soft transparent tissue guard appliance for each patient. (Fig. 3) This customized appliance was made of a clear acrylic plastic material (SPLINT PVC sheets – China). These sheets available in two forms: hard and soft readymade transparent thermoplastic sheets with different thickness. In this study we used the soft form with 1.5 mm thickness. The sheets are biocompatible and already found in the market worldwide and used by many orthodontists for retainer fabrication and by many dentists for night guards and whitening trays fabrication. The appliance used in the current study is a modification in the design of the soft night guard with extended borders to cover the wound site. No adverse side effects were noticed throughout the study and no complaints were reported by all participants.

The design and the use of soft tissue guard appliance is an exclusive idea used in our study. The guard was designed to be soft and tissue biocompatible, yet, rigid enough to prevent its distortion while in function. It was used to achieve maximum HA gel absorption by containing and keeping the gel in intimate contact with the tissues, preventing its washing out quickly after application and to prevent mechanical trauma to the wound site during the first week of the healing phase.

**Fig. 4:**
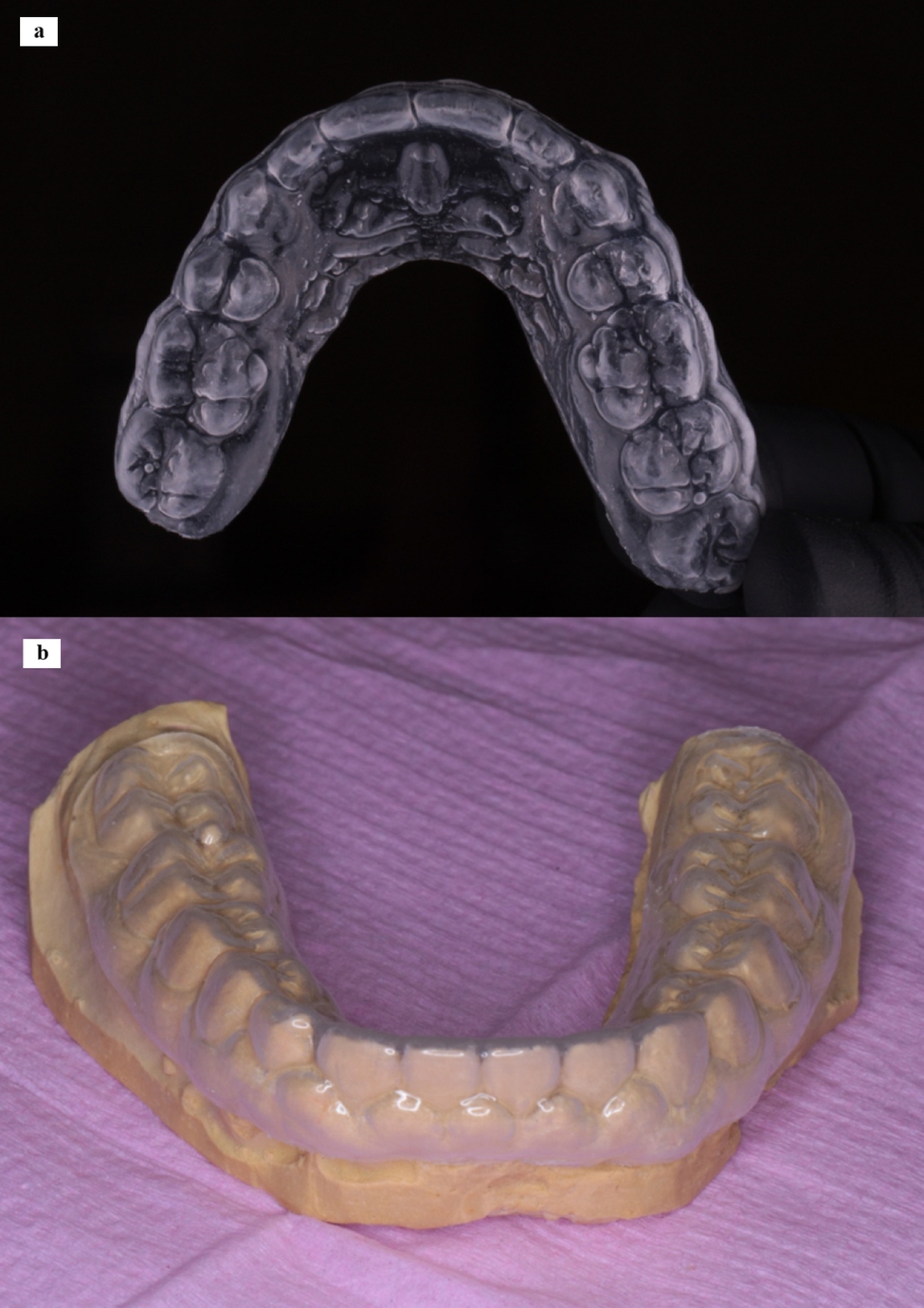
Custom-made transparent soft tissue guard appliance. **a**. appliance made for upper arch, **b**. appliance made for lower arch

Similar to the removable retainers and night guards, the used soft tissue guard appliance has excellent patient handling and seating properties. The margins of the appliance were extended enough to cover the wound site after surgery, meanwhile not sharp or irritating the oral mucosa. (Fig. 4)

**For group II;** after hemostasis, the surgical site of the test group was subjected to laser irradiation using diode laser immediately after surgery and at days 3,7 and 14. The delivery tip was perpendicular to the gingival tissue surface at 1 cm distance with the same PBMT parameters as in group I.

### Post-surgical care

Immediately following the procedure, an ice pack was recommended to the patient, and post-operative instructions were given (both written and verbal). A standardized nonsteroidal anti-inflammatory (Diclofenac potassium 50 mg) was prescribed. They were instructed to take the medicine only if required, maximum two times a day for five days. Patients in the test group were instructed to wear the soft tissue guard appliance lined by HA for the first week to cover the exposed wound site and to keep the HA gel in close contact with the tissues for longer time. They were advised to remove the tissue guard appliance only before eating and consume only soft food during the first week to avoid any mechanical trauma. Patients were instructed to brush their teeth carefully with soft brush away from the wound site. As well as brushing the inner surface of the guard to clean it before gel re-application. No mouth wash was prescribed to avoid confounding variable and to exclude any external factor that can affect the healing. Patients were recalled after 3, 7 and 14 days for post-operative follow-up and for PBMT and HA re-application, then at day 21 for final assessment.

### Patient’s assessment

Following the surgical gingivectomy procedure, the wound healing was assessed post PBMT using the healing index by Landry et al. [35] (Table 2) which grades the wound on a scale of 1–5, where 1 indicates very poor healing and 5 indicates excellent healing. Each grade contains 4 important parameters to be assessed as shown in Table 2. These parameters were recorded every follow up day either clinically to assess the bleeding on palpation or digitally by taking standardized photographs to assess other parameters like tissue color change and epithelization. These digital images were obtained immediately after the gingivectomy procedure and on all the following visits with the same camera settings, same distance and same source of light (Canon DSLR 80 D (shutter speed = 1/125, F = 22, ISO = 250), Canon EF100mm macro lens (on scale of 3:1, 0.48 m) and Yongnuo YN-14EX LED macro ring flash light with intensity of 1/4). A 1 mm × 1 mm digital grid was superimposed onto the digitized images to standardize all the clinical photographs to make sure that they are all on the same scale. Tissue color change between the day of the surgery and post surgically (Day 3.7.14.21) from red to pink was assessed from the clinical photographs on ImageJ software. The wound surface area was calculated in pixels by the same software for assessment of wound surface area epithelization. All measurements and scores were recorded by one calibrated examiner who was not informed about the group of which the participant was assigned to.

**Table 2:** Landry index for soft tissue healing [35]

Healing grade	Clinical criteria
very poor (1)	Tissue color: ≥50% of gingiva red Response to palpation: Bleeding Granulation tissue: Present Incision margin: Not epithelialized, with loss of epithelium beyond incision margin.
Poor (2)	Tissue color: ≥50% of gingiva red Response to palpation: Bleeding Granulation tissue: Present Incision margin: Not epithelialized, with connective tissue exposed
Good (3)	Tissue color: ≥25% and < 50% of gingiva red Response to palpation: No bleeding Granulation tissue: None Incision margin: No connective tissue exposed
Very good (4)	Tissue color: <25% of gingiva red Response to palpation: No bleeding Granulation tissue: None Incision margin: No connective tissue exposed
Excellent (5)	Tissue color: All tissues pink Response to palpation: No bleeding Granulation tissue: None Incision margin: No connective tissue exposed

Patients were instructed to chart their perceptions of pain using a visual analog scale (VAS) with a range of 0 (no pain/burning sensation) to 10 (severe pain/burning sensation) for 21 days starting from the day of the operation [36]. All participants were given a printed copy that contained 21 scale and a guide shows face expressions for each score. Also, to standardize the time of recording, the patients were instructed to record the score every day at specific time with the help of a mobile phone reminder.

### Statistical analysis

Descriptive statistics were calculated as means, standard deviation (SD), medians, interquartile range (IQR), frequencies and percentages. Comparisons between the two study groups were performed using Mann-Whitney U test, while comparisons between different timepoints within each group were performed using Friedman test, followed by multiple pairwise comparisons using Bonferroni adjusted significance level. Significance was set at p value < 0.05. Data were analyzed using IBM SPSS for Windows (Version 26.0).

## Results

Figure 1 (consort flow chart) shows that out of the total 36 patients assessed for eligibility, only 26 were included in the current study. Table 3 shows that there were no significant differences in the demographic characteristics of both groups. The majority of participants were females with mean (SD) age = 22.31 (2.93) and 22.46 (2.82) in the test and control groups, respectively.

**Table 3:** Showing age and gender distribution in the two study groups

		Test (n = 13)	Control (n = 13)	Total	P value
Age	Mean (SD)	22.31 (2.93)	22.46 (2.82)	22.38 (2.82)	0.89
Gender: n (%)	Male	3 (23.1%)	5 (38.5%)	8 (30.8%)	P_FE_: 0.67
	Female	10 (76.9%)	8 (61.5%)	18 (69.2%)	

Age and gender were compared using independent samples t-test and Fisher exact test, respectively

Table 4 represents comparison of Landry’s healing index in the two study groups. On the third day, 6 patients in the test group showed good healing (46.2%) compared to 2 patients in the control group (15.4%). Meanwhile, the test group showed significantly higher healing scores on days 7, 14, and 21 (p = 0.03, 0.02, and 0.006, respectively). Comparisons highlighted that excellent healing score 5 on Landry healing index (38.5%) started on day 14 in test group (p < 0.001). By the end of the follow-up period, the test group showed excellent healing (100%) in all the cases compared to only 5 cases in the control group showing excellent healing (38.5%) and 8 cases showing very good healing (61.5%) (p = 0.006).

**Table 4:** Comparison of Landry Healing Index scores between the two study groups at days 3, 7, 14 and 21 and post –hoc analysis of these values at the different study time points

		Test (n = 13)	Control (n = 13)	MWU P value
Day 3	Very poor (1)	0 (0%)	0 (0%)	0.10
	Poor (2)	7 (53.8%)	11 (84.6%)	
	Good (3)	6 (46.2%)	2 (15.4%)	
	Very good (4)	0 (0%)	0 (0%)	
	Excellent (5)	0 (0%)	0 (0%)	
	Mean (SD)	**2.46 (0.52)**	**2.15 (0.38)**	
	Median (IQR)	**2.0 (2.0, 3.0)**	**2.0 (2.0, 2.0)**	
Day 7	Very poor (1)	0 (0%)	0 (0%)	0.03*
	Poor (2)	0 (0%)	0 (0%)	
	Good (3)	7 (53.8%)	12 (92.3%)	
	Very good (4)	6 (46.2%)	1 (7.7%)	
	Excellent (5)	0 (0%)	0 (0%)	
	Mean (SD)	**3.46 (0.52)**	**3.08 (0.28)**	
	Median (IQR)	**3.0 (3.0, 4.0)**	**3.0 (3.0, 3.0)**	
Day 14	Very poor (1)	0 (0%)	0 (0%)	0.02*
	Poor (2)	0 (0%)	0 (0%)	
	Good (3)	0 (0%)	0 (0%)	
	Very good (4)	8 (61.5%)	13 (100%)	
	Excellent (5)	5 (38.5%)	0 (0%)	
	Mean (SD)	**4.38 (0.51)**	**4.00 (0.00)**	
	Median (IQR)	**4.0 (4.0, 5.0)**	**4.0 (4.0, 4.0)**	
Day 21	Very poor (1)	0 (0%)	0 (0%)	0.006*
	Poor (2)	0 (0%)	0 (0%)	
	Good (3)	0 (0%)	0 (0%)	
	Very good (4)	0 (0%)	8 (61.5%)	
	Excellent (5)	13 (100%)	5 (38.5%)	
	Mean (SD)	**5.00 (0.00)**	**4.38 (0.51)**	
	Median (IQR)	**5.0 (5.0, 5.0)**	**4.0 (4.0, 5.0)**	
Friedman test p value		< 0.001*	< 0.001*	
Post-hoc test	Day 3 compared to 7	0.17	0.35	
	Day 3 compared to 14	< 0.001*	< 0.001*	
	Day 3 compared to 21	< 0.001*	< 0.001*	
	Day 7 compared to 14	0.35	0.07	
	Day 7 compared to 21	0.005*	0.005*	
	Day 14 compared to 21	0.89	1.00	

MWU: Mann-Whitney test was used

*statistically significant at p value < 0.05

Post-hoc comparisons were performed using Bonferroni adjusted significance level

**Fig. 5:**
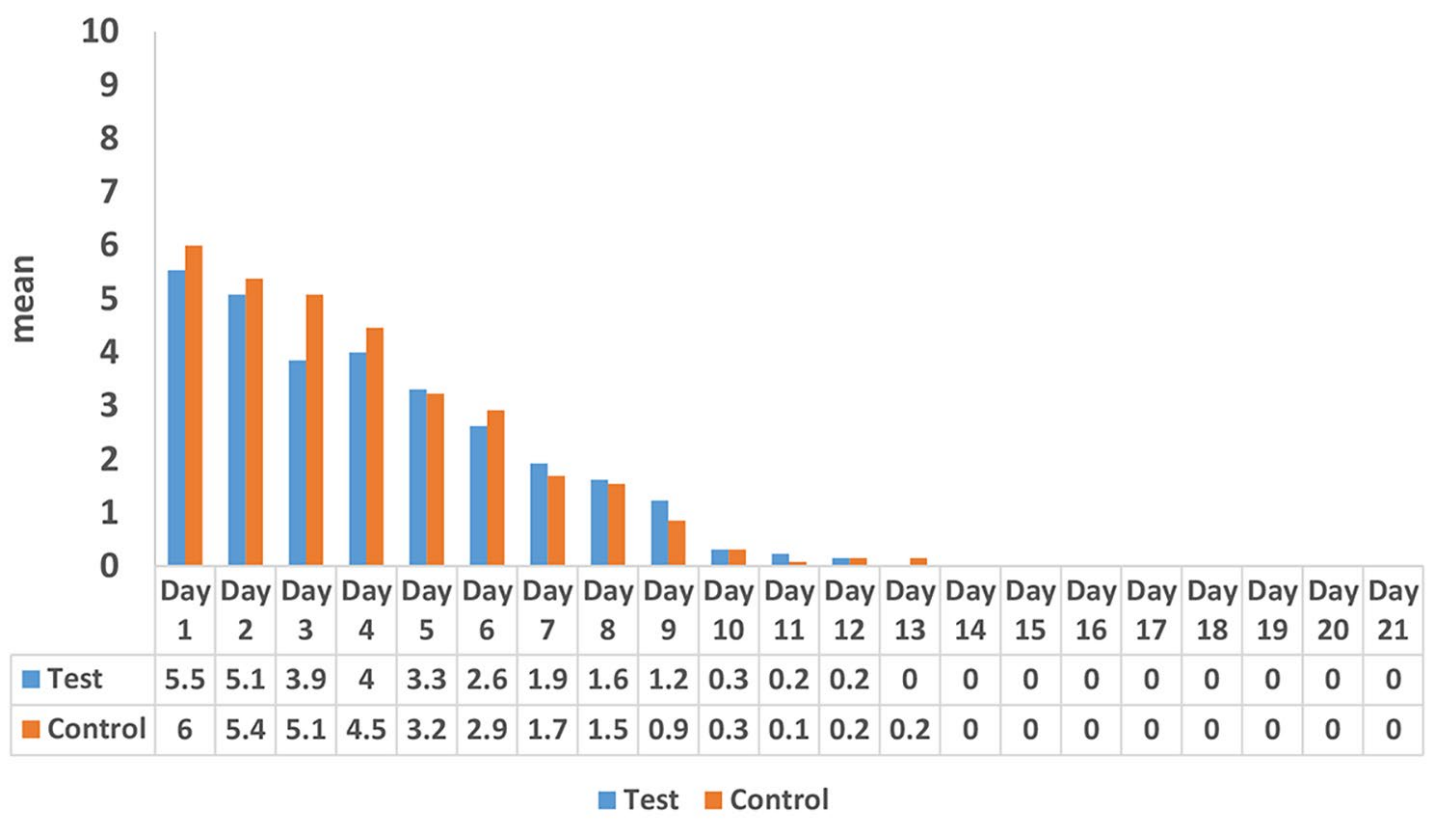
Perceived pain using VAS at different time points in the two study groups

Figure 5 shows the perceived pain intensity using VAS over the study period in the two study groups. There were no significant differences in VAS scores between both groups (p > 0.05) with slightly higher scores in the control group. Within group comparisons showed that pain improved significantly starting from day 9 (p = 0.008 and 0.001 in the test and control groups, respectively).

## Discussion

Wound healing after surgical gingivectomy occurs by secondary intention [5]. During healing by secondary intention, wound can be associated with discomfort and delayed healing compared to primary intention healing wounds. To enhance the healing process and shorten its duration, the application of photobiomodulation (PBM) as an adjunctive therapy has attracted the attention of many researchers in recent years [13]. Many studies showed that PBMT was an effective adjunctive treatment that appeared to promote healing following gingivectomy [37–39]. Besides PBMT, the use of many other topical therapeutic agents have been reported in the literature to improve the wound healing after gingivectomy procedure such as: hyaluronic acid (HA) gel, herbal gel, non-thermal atmospheric pressure plasma application and vitrocure ® gel [2, 40, 41].

To the best of our knowledge, no study to date has examined the effect of PBMT in combination with pure HA gel on wound healing after gingivectomy. The latter was used in the current study as recently, there has been a surge in the use of HA for cosmetic and medical reasons. In 2022, a systemic review by Rodríguez-Aranda et al. [42] demonstrated favorable results with HA in periodontal regeneration. In the field of periodontology, HA has been advocated as monotherapy [43] or as an adjunct to non-surgical [44], surgical [45] or laser-assisted periodontal treatment [46] to reduce inflammation and promote wound healing. All of these studies used the commercially available HA gel and its highest concentration used was 0.8%, while no study has used pure HA gel with a higher concentration as used in our study. Moreover the advantage of examining pure HA gel is to determine its synergistic effect without any additives that found in the commercially available HA gel preparations so, excluding any confounder variables.

The effect of HA on wound healing can be explained based on its properties that acts to promote soft tissue healing. One of the main known properties of HA that it has an.

anti‑inflammatory effect [17]. There is also evidence that extracellular matrix remodeling following application of HA matrices is enhanced and collagen deposition is more ordered with less degradation [47]. To summarize the function of HA during wound healing there are three phases [48]: First, in the inflammatory phase where HA allows inflammatory cell migration. Second, in the proliferative phase where HA draws fibroblasts to wound site, promotes keratinocyte migration and proliferation. Third, in the remodeling phase: HA contributes to promote normal healing and to increase wound strength progressively.

**Fig. 6:**
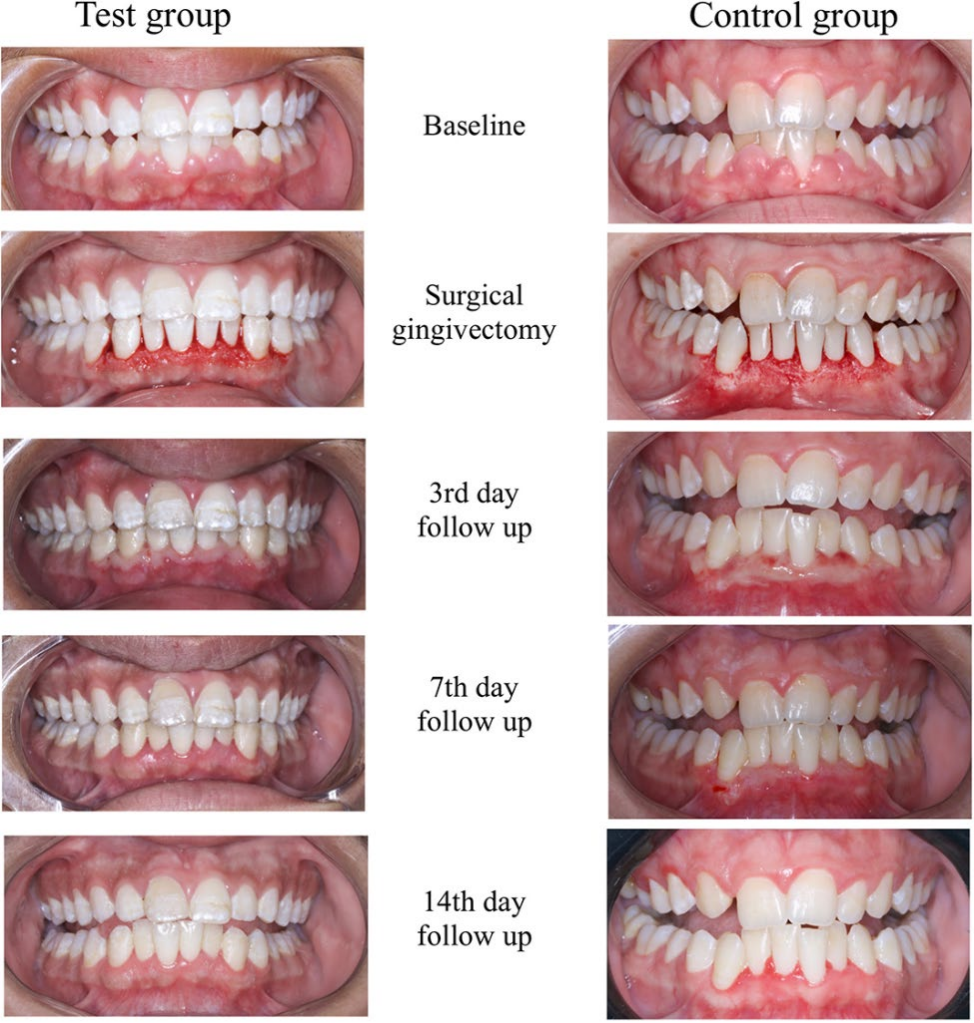
Wound healing after surgical gingivectomy in the two study groups

Our findings regarding wound healing assessment by Landry Healing Index showed that application of HA and PBMT in the test group positively promoted wound healing following surgical gingivectomy procedure (Fig. 6) as well as decreased post-operative pain perception by patients (Fig. 5). This finding was in line with the results of a randomized clinical trial conducted by Lingamaneni et al. [49] investigating the effects of PBM therapy on wound healing after gingivectomy. Authors observed better soft tissue healing and surface epithelialization after 14 days. Moreover, in accordance to our results, Mahmoud et al. [11] examined PBM therapy on wound healing post gingivectomy using laser of wavelength 850 nm for 4 sessions on day 0, 3, 7 and day 14 post gingivectomy. Wound healing was assessed using Landry Healing Index, and similarly, the results of their study supported that the PBMT was significantly effective (p < 0.001) on wound healing after gingivectomy.

Furthermore, in accordance to our findings Turgut Çankaya et al. [46] evaluated the effect of application of HA following laser-assisted frenectomy. Authors concluded that HA was a viable option for decreasing the surface area of the wound within 14 days and act as a wound dressing following frenectomy. The soft tissue healing potential of HA in our study may be explained based on a histological study conducted by Reddy et al. [2], who concluded that HA gel (0.2%) has an anti-inflammatory properties and has induced more epithelial tissue formation and increased connective tissue vascular supply, histologically.

In the current study, the addition of pure 2% HA gel after PBMT exhibits a positive impact on wound healing acceleration as shown in Table 4. The test group showed significantly higher healing scores on days 7, 14, and 21 (p = 0.03, 0.02, and 0.006, respectively). Yıldırım et al. [20] used 0.2% and 0.8% HA gel for accelerating palatal wound healing. The results of this study revealed that complete epithelization of palatal wound was achieved on day 21 in both test groups. Earlier wound healing in the current clinical trial may be attributed to the higher concentration of HA gel we used.

Uslu and Akgül [1] assessed the PBM and ozone application after gingivectomy and gingivoplasty. VAS pain results obtained in their study were significantly lower in PBM and ozone groups. Whereas in the current study, over the study period in the two study groups, there were no significant differences in VAS scores between both groups with slightly higher scores in the control group. This could be explained primarily based on the fact that VAS assessment is an objective method and pain threshold is not the same for all patients. Furthermore, the standardized PBMT parameters and postoperative care (NSAID) may also explain our non-significant differences in VAS.

Contradictory to our results, Masse et al. [50] reported that PBM therapy applied after periodontal surgeries using soft laser showed no significant differences in the gingival index, healing index and pain reduction. They evaluated the postoperative pain by modified McGill pain scale. This scale, which consists of 20 main parts with 6 different pain levels, is more complex and difficult to be followed by patients than VAS scale. In addition, the different laser type may explain the possible contradictory results. Also, on the contrary to our results, Damante et al. [51] showed that PBMT using 15-mW diode laser (670 nm wavelength) did not accelerate the healing of oral mucosa after gingivoplasty. We believe that these different findings in the literature may be due to many reasons such as differences in the laser device selected, laser parameters, application times, soft tissue healing scores and the type of treated tissues.

Small sample size and lack of histological evaluation of wound healing can be considered as limitations of the study. Within the limitations of the present study, PBM and 2% HA applications after surgical gingivectomy improved the quality of life of the patients. Further clinical trials with larger populations are recommended to assess the synergistic effect of PBM and HA application on wound healing in different periodontal surgical procedures. Moreover, histological assessment of soft tissue healing is also recommended.

## Conclusions

Photobiomodulation therapy (PBMT) can improve wound healing after surgical gingivectomy, but application of (2%) high molecular weight hyaluronic acid gel as an adjunctive to PBMT was found to have significant clinical effects and higher power of repair among test group when compared to that achieved by PBMT alone in control group. Within the limitation of the study, it could be concluded that HA gel improved the healing outcomes by decreasing the time required for complete wound enclose and re-epithelialization.

## Data Availability

All data included in this study are available from the corresponding author upon request.

## List of abbreviations

LLLT: Low level laser therapy
PBMT: Photobiomodulation Therapy
PBM: Photobiomodulation
PMPR: Professional mechanical plaque removal
HA: Hyaluronic acid
HMW: High molecular weight
NSAID: Nonsteroidal anti-inflammatory drugs
VAS: Visual Analog scale
